# Current status of education and research on public health nutrition in Japan: comparison with South Korea, Taiwan, and mainland China

**DOI:** 10.1186/s40795-019-0275-x

**Published:** 2019-02-12

**Authors:** Nana Shinozaki, Han-Chieh Wang, Xiaoyi Yuan, Tianyu Li, Kana Asano, Satomi Kobayashi, Satoshi Sasaki

**Affiliations:** 1 Department of Social and Preventive Epidemiology, Graduate School of Medicine, The University of Tokyo, Tokyo, Japan; 2 Department of Food and Nutrition, College of Human Ecology, Seoul National University, Seoul, South Korea; 3 Department of Social and Preventive Epidemiology, School of Public Health, The University of Tokyo, 7-3-1 Hongo, Bunkyo-ku, Tokyo, 113-0033 Japan

**Keywords:** Public health nutrition, Community nutrition, Education, East Asia, Japan

## Abstract

**Background:**

Although the importance of capacity building for public health nutrition (PHN) has been increasing globally, reports on the current status of training programs for PHN in East-Asia including Japan are limited. The aim of this study was to compare the current status of education and research activities in the field of PHN in Japan with those in South Korea, Taiwan, and mainland China.

**Methods:**

Necessary information was collected by internet search and telephone inquiry. Collection focused on the number of departments in colleges and universities with PHN as a compulsory subject in the 2016 academic year, and the number of articles and information related to these articles published in the journal *Public Health Nutrition* between 2007 and 2016.

**Results:**

The number of departments with PHN as a compulsory subject was the highest in Japan (*n* = 137), followed by mainland China (*n* = 32), Taiwan (*n* = 18) and South Korea (*n* = 7). Using the classification list of education in each country and region, the majority of these departments were classified as home economics, natural science, health and welfare, and medical science in Japan, South Korea, Taiwan, and mainland China, respectively. Regarding publications, most of the articles were written in colleges and universities not having PHN as a compulsory subject in Japan, South Korea, and Taiwan. The number of articles per department among departments with compulsory PHN education was lowest in Japan (*n* = 0.3) compared to Taiwan, mainland China, and South Korea (*n* = 1.2, 2.7, and 3.7, respectively).

**Conclusions:**

Japan has a much higher number of departments with PHN as a compulsory subject than neighboring East Asian states and relatively low research activities in the field of PHN. This suggests that current university education may not lead to active PHN research in Japan. Further studies are warranted to explore the reasons for this.

## Background

Poor nutrition plays a major role in the development of lifestyle-related diseases such as obesity, type 2 diabetes, and cardiovascular diseases [1]. To date, various health goals and policies have been established to tackle nutritional issues and many health promotion interventions have been implemented [1, 2]. Effective public health nutrition (PHN) practice requires a skilled workforce, and recognition of the importance of capacity building for PHN has increased globally [2–10]. In recent years, many countries have developed a variety of training programs aimed at fostering highly specialized human resources for PHN, such as online courses, global master’s programs, and summer schools, with examples in the US, Australasia, Middle East, Africa, and Europe [2, 8–11].

Meanwhile, only a few reports have described the current status of training programs for PHN in Asia, and several of these have indicated insufficiencies in education [12–18]. For instance, PHN has not been set as an independent academic discipline in any college or university in India or Vietnam [13, 14]. Research on postgraduate education in South Asian countries found that 5 of 8 surveyed countries had neither nutrition nor PHN as an academic discipline at the master’s level [15]. In Japan, educational content or teaching skills for PHN in colleges and universities has been described as insufficient [17, 18].

Historically, nutrition science, including PHN, has not been regarded as an important academic discipline in Japan [19, 20]. A previous study reported that 7 of 9 prominent colleges or universities that published articles on human nutrition research did not have a department or division of nutrition, and that universities for training researchers in human nutrition were not sufficient in either quality and in quantity [19]. In particular, PHN, which is characterized as macroscopic science, was considered to have a relatively low status among academic disciplines because the development of science in Japan has focused on microscopic research [18]. Given this, the status of education and research in PHN may be particularly prejudiced. To our knowledge, however, no study has yet evaluated PHN education in Japan from a quantitative standpoint, despite the fact that the future development of PHN requires assessment of the current status of education and research. We considered that the current status of education and research in PHN in Japan could be better understood by comparison with neighboring states in the East Asian region, namely South Korea, Taiwan, and mainland China, which have much in common with Japan and also lack data on the current status of PHN.

Here, to better understand the current status of education and research of PHN in East Asia, including Japan, we compared the number of colleges or universities offering PHN education in Japan and South Korea, Taiwan, and mainland China, as well as the educational fields of the departments and the number of journal articles and research institutions of PHN.

## Methods

### Number of educational institutions and departments of PHN

From December 2016 to July 2017, we conducted a situational analysis through a systematic internet search to identify institutions and departments offering PHN education across Japan, South Korea, Taiwan, and mainland China (hereafter China). We searched for school information in the 2016 academic year; if this was unavailable, we used the latest available information prior to this year. This study targeted colleges and universities that confer degrees equivalent to bachelor’s degree. In 2016, there were 775, 229, 158 and 1236 colleges or universities in Japan, South Korea, Taiwan, and China, respectively [21–24]. In China, however, since many schools did not make official information available online, we investigated only the 112 key national colleges and universities designated in “Project 211” [25], which were considered to have relatively high reliability and availability of school information. Educational institutions in Hong Kong and Macao were not included in that project. We also excluded distance learning schools (*n* = 4, 18, and 2 in Japan, South Korea, and Taiwan, respectively) and military schools (*n* = 7 and 3 in Taiwan and China, respectively), as well as 16 schools in China whose school website was not accessible or phone number was not available. After these exclusions, the total number of colleges or universities was 771, 211, 149, and 93 in Japan, South Korea, Taiwan, and China, respectively.

We then identified departments at these colleges and universities which offered PHN education. First, PHN classes were sought using syllabus, course guidelines, and school regulations on the school websites. PHN classes were defined as those having words that mean “public health nutrition” or “community nutrition” in the local languages of each country or region in the class name. In China, since few classes met this condition, classes covering the topic of PHN (“nutrition and food safety” and “food and nutrition”) were also considered to define PHN classes. Second, we identified departments that required a PHN class as a compulsory subject (i.e., PHN program). When we could not obtain sufficient information on PHN class or department on the school website, we made telephone inquiries to the school. In Taiwan, remedial education courses and continuing education courses were excluded because their educational systems were different to those of general courses. In China, since information was unavailable for most departments, our investigation of PHN programs was restricted to departments that were likely to have a PHN class, determined as follows. Considering that PHN is conceptually related with nutrition or food, we chose departments with the word “nutrition” or “food” in their name. We also chose departments at faculties of medicine on the basis that nutrition is educationally classified in China under medicine [26].

### Educational field of the departments of PHN

To clarify the educational field of PHN, we classified the PHN programs by field of education using the official classification methods of each country or region. In Japan, South Korea, and China, departments were classified into 12, 7, and 12 fields of education, respectively, by the name of the department [26–28]. In Taiwan, departments were classified into 9 fields of education based on the major subject of the department [29]. In the case that one department is categorized as having an overlapping plurality of fields of education under the Japanese classification method, the reciprocal of the number of overlapping fields was distributed to the corresponding fields. A department having both a 2-year and a 4-year PHN program was counted as one.

### Number of journal articles and research institutions of PHN

To identify research institutions of PHN, we investigated PHN-related journal articles published by an author who belonged to a domestic institution of each country and region over the last 10 years and the author’s affiliated institution. First, we conducted a literature search through PubMed in January 2017 to identify articles published in *Public Health Nutrition* from 2007 to 2016. The search strategy in the survey of Japan was as follows: public health nutrition [Journal] AND “Japan”[All Fields] AND (“2007/01/01”[PDAT]: “2016/12/31”[PDAT]). The word “Japan” was changed to “Korea”, “Taiwan” or “Republic of China”, and “China” in the survey of South Korea, Taiwan, and China, respectively. Articles published from institutions in Hong Kong and Macao were excluded. Next, we examined affiliated institutions of the first and corresponding authors who belonged to a domestic institution of the respective country or region. First author was defined as the person whose name was written at the beginning of an author list and corresponding author as the person who was marked as a “corresponding author” in an article. If an author had more than one affiliation, the first affiliation listed was regarded as the author’s affiliation. Affiliated institutions were classified as follows: educational institution (college, university or graduate school) with a PHN program; educational institution without a PHN program; hospital (including university hospitals) or research institute (including research centers of the university); and other institution. A graduate school that had a PHN program at its affiliated department was regarded as an educational institution with a PHN program.

We calculated the number of PHN-related articles published from each type of affiliated institution for the first and corresponding author in each country and region. When one article was written by plural first or corresponding authors, the reciprocal of the number of first or corresponding authors of the article was distributed to their affiliated institutions. We also calculated the number of PHN-related articles per PHN program in each country and region by dividing the total number of PHN-related articles (written by a first or corresponding author who belonged to one of the identified domestic institutions) by the total number of PHN programs of each country or region. Additionally, to remove the influence of the productivity of a specific author, we counted the number of affiliated institutions by the type of institution in each country and region, regardless of the number of articles they published. Affiliated institutions were classified as faculty department or graduate school according to their name. All calculations were performed using Microsoft Excel 2010.

## Results

The number of colleges and universities surveyed is shown in Table 1. Course information was not available for 18 institutions in China because their website did not offer the information and phone calls could not be connected. Japan had the largest number of colleges or universities with PHN programs. In Japan, South Korea, and Taiwan, private schools provided more PHN programs than national schools.

**Table 1:** Number of colleges and universities surveyed in this study

	Japan		South Korea		Taiwan		China^†^	
	n	%^‡^	n	%^‡^	n	%^‡^	n	%^‡^
Total number of the colleges and universities	771		211		149		93	
National schools	86	11	45	21	47	32	70	75
Public schools	88	11	1	1	1	1	23	25
Private schools	597	77	165	78	101	68	0	0
Total number of the colleges and universities with a PHN program^§^	128		7		18		24^¶^	
National schools	5	4	1	14	1	6	14	58
Public schools	18	14	0	0	0	0	10	42
Private schools	105	82	6	86	17	94	0	0

^†^Only colleges and universities whose school website, email address, or phone number was available, and those designated in “Project 211” were investigated in this study

^‡^Some percentages do not total 100 because of rounding

^§^A PHN program was defined as a department requiring a class in which the class name included words that meant “public health nutrition” or “community nutrition” in the language of the respective country or region. In China, the class named “nutrition and food safety” or “food and nutrition” were also defined as PHN classes

^¶^Eighteen colleges or universities were not included because information on whether or not PHN classes were compulsory was not available

The number of PHN programs and their fields of education as classified using the classification method of each country and region are shown in Table 2. The total number of PHN programs was 137, 7, 18, and 32 in Japan, South Korea, Taiwan, and China, respectively. The most dominant field of education of the PHN programs was home economics in Japan, natural science in South Korea, health and welfare in Taiwan, and medical science in China.

**Table 2:** Fields of education of PHN programs^†^ classified by the official classification methods

Japan			South Korea			Taiwan			China^‡^		
Field of education^§^	n^¶^	%^♯^	Field of education^§^	n	%	Field of education	n	%	Field of education^§^	n	%
Home economics	89.9	66	Natural science	4	57	Health and welfare	15	83	Medical science	23	72
Health (not medical science or dentistry)	24.1	18	Medical science and pharmacy	3	43	Agriculture	2	11	Engineering	9	28
Unspecified	10.9	8	Social Science	0	0	Education	1	6	Philosophy	0	0
Agriculture	9.2	7	Arts and physical education	0	0	Humanities and arts	0	0	Economics	0	0
Education	2.3	2	Humanities	0	0	Social sciences, business and law	0	0	History	0	0
Humanities	0.3	0	Engineering	0	0	Science	0	0	Law	0	0
Social science	0.3	0	Education	0	0	Engineering, manufacturing and construction	0	0	Education	0	0
Natural science	0.0	0				Services	0	0	Literature	0	0
Engineering	0.0	0				Unspecified	0	0	Natural science	0	0
Health (medical science & dentistry)	0.0	0							Agriculture	0	0
Merchant marine	0.0	0							Management and administration	0	0
Arts	0.0	0							Arts	0	0
Total	137	100	Total	7	100	Total	18	100	Total	32	100

^†^A PHN program was defined as a department requiring a class in which the class name included words that meant “public health nutrition” or “community nutrition” in the language of the respective country or region. In China, the class named “nutrition and food safety” or “food and nutrition” were also defined as PHN classes

^‡^Only colleges and universities whose school website, email address, or phone number was available, and those designated in “Project 211” were investigated in this study

^**§**^The name of the field was translated by the authors

^¶^Values are represented including the decimal point because some departments were categorized into overlapping plurality of fields of education under the Japanese classification method. Numbers for these were calculated by distributing the reciprocal of the number of overlapping fields to the corresponding fields

^♯^The percentage does not total 100 because of rounding

Of articles published in *Public Health Nutrition* from 2007 to 2016, the number of articles accessed with each search term was 50, 33, 21, and 88 in Japan, South Korea, Taiwan, and China, respectively. The number of articles written by a first or corresponding author who belonged to a domestic institution in each of the states and published in *Public Health Nutrition* from 2007 to 2016 is shown in Table 3. The results for first author were nearly identical to those for corresponding author. The number of PHN-related articles written by a first author was 41, 26, 21, and 86 in Japan, South Korea, Taiwan, and China, respectively. In all the states, educational institutions produced more PHN-related articles than hospitals, research institutes, and other institutions. In Japan, South Korea, and Taiwan, educational institutions with a PHN program produced fewer articles than those without a PHN program. The ratio of articles written in hospitals to all articles was highest in South Korea and lowest in Japan. The number of PHN-related articles written by a first author who belonged to an identified institution in each country and region per PHN program of each country and region was lowest in Japan (*n* = 0.3), followed by Taiwan (*n* = 1.2), China (*n* = 2.7), and South Korea (*n* = 3.7).

**Table 3:** Number of PHN-related articles^†^ published from 2007 to 2016

	First author								Corresponding author							
	Japan		South Korea		Taiwan		China		Japan		South Korea		Taiwan		China	
	n	%^‡^	n	%^‡^	n	%^‡^	n	%^‡^	n	%^‡^	n	%^‡^	n	%^‡^	n^§^	%^‡^
Total number of articles	41		26		21		86		42		26		21		82	
Affiliated institutions																
Educational institution^¶^	33		13		13		37		33		13		13		36.3	
With PHN program^#^	10	24	0	0	5	25	26	30	9	21	0	0	4	19	27.3	33
Without PHN program	23	56	13	50	7	35	4	5	24	57	13	50	9	43	4.0	5
Unknown^††^	0	0	0	0	0	0	7	8	0	0	0	0	0	0	5.0	6
Hospital^‡‡^	1	2	9	35	3	15	16	19	1	2	9	35	2	10	12.2	15
Research institute^§§^	7	17	4	15	4	20	22	26	8	19	4	15	6	29	25.5	31
Other^¶¶^	0	0	0	0	1	5	11	13	0	0	0	0	0	0	8.0	10
No. of articles per PHN program^##^	0.3		3.7		1.2		2.7		0.3		3.7		1.2		2.6	

^†^Articles written by a first or a corresponding author who belonged to a domestic institution of Japan, South Korea, Taiwan, or China, and published in *Public Health Nutrition*

^‡^Some percentages do not total 100 because of rounding

^§^Values include the decimal point because the number of articles written by plural corresponding authors was calculated by distributing the reciprocal of the number of corresponding authors of the article to each institution

^¶^College, university and graduate school

^#^A PHN program was defined as a department requiring a class in which the class name included words that meant “public health nutrition” or “community nutrition” in the language of the respective country or region. In China, the class named “nutrition and food safety” or “food and nutrition” were also defined as PHN classes. A graduate school which has a PHN program at its affiliated department was regarded as an educational institution with a PHN program

^††^Educational institutions whose school website could not be accessed from Japan or whose phone number was unavailable

^‡‡^Hospital included university hospitals

^§§^Research institute included research centers of the university

^¶¶^Other institutions included companies, international organizations, and centers for disease control and prevention

^##^No. of articles per PHN program = the total number of articles that were written by the first or corresponding author who belonged to each domestic institution, published in *Public Health Nutrition* from 2007 to 2016/number of PHN programs in each country or region

The number of institutions that published PHN-related articles in *Public Health Nutrition* from 2007 to 2016 is shown in Table 4. Results for first author and corresponding author were closely similar. The number of institutions for first authors was 24, 19, 15, and 53 in Japan, South Korea, Taiwan, and China, respectively. In all the states, the number of educational institutions was largest of all institutions. In Japan, South Korea, and Taiwan, the number of educational institutions with a PHN program was less than that without a PHN program. Similarly to the result for the number of articles, the proportion of hospitals to all institutions was highest in South Korea and lowest in Japan.

**Table 4:** Number of affiliated institutions of authors who published PHN-related articles from 2007 to 2016^†^

	First author								Corresponding author							
	Japan		South Korea		Taiwan		China		Japan		South Korea		Taiwan		China	
	n	%^‡^	n	%^‡^	n	%^‡^	n	%^‡^	n	%^‡^	n	%^‡^	n	%^‡^	n	%^‡^
Total number of affiliated institutions	24		19		15		53		24		21		11		49	
Educational institution^§^	18		9		10		19		18		11		8		17	
With PHN program^¶^	4	17	0	0	4	27	11	21	5	21	0	0	3	27	10	20
Without PHN program	14	58	9	47	6	40	4	8	13	54	11	52	5	46	4	8
Unknown^#^	0	0	0	0	0	0	4	8	0	0	0	0	0	0	3	6
Hospital^††^	1	4	6	32	3	20	15	28	1	4	6	29	2	18	12	25
Research institute^‡‡^	5	21	4	21	1	7	10	19	5	21	4	19	1	9	14	29
Other^§§^	0	0	0	0	1	7	9	17	0	0	0	0	0	0	6	12

^†^Affiliated institutions of first or corresponding authors who belonged to a domestic institution of Japan, South Korea, Taiwan, or China, and published an article in *Public Health Nutrition*. The number of each type of institution was calculated regardless of the number of articles they have published. Affiliated institutions were classified by the name equivalent to the faculty or graduate school

^‡^Some percentages do not total 100 because of rounding

^§^College, university and graduate school

^¶^A PHN program was defined as a department requiring a class in which the class name included words that meant “public health nutrition” or “community nutrition” in the language of the respective country or region. In China, the class named “nutrition and food safety” or “food and nutrition” were also defined as PHN classes. A graduate school which has a PHN program at its affiliated department was regarded as an educational institution with a PHN program

^#^Educational institutions whose school website could not be accessed from Japan or whose phone number was unavailable

^††^Hospital includes university hospitals

^‡‡^Research institute includes research centers of a university

^§§^Other institutions include companies, international organizations, and centers for disease control and prevention

## Discussion

In this study, we found that the dominant field of education of PHN programs was different among Japan, South Korea, Taiwan, and China. Japan had by far the largest number of PHN programs, whereas the number of PHN-related articles per PHN program was lowest in Japan. This finding suggests that PHN education may not lead to active PHN research in Japan. To our knowledge, this is the first study to compare the current status of PHN education and research in Japan with those in South Korea, Taiwan, and China.

The most dominant field for PHN education under the Japanese classification was home economics, a field which did not exist in the classifications of South Korea, Taiwan, or China. With regard to university education background, nutrition was taught in departments of home economics at the time when home economics was established in higher education in all the states [30–34]. In South Korea, Taiwan, and China, however, the name ‘home economics’ was changed or nutrition became independent from departments of home economics during the process of postwar educational reform and specialization and differentiation among academic disciplines [33–35]. Meanwhile, in Japan, home economics was established as an academic discipline in higher education during the educational reforms which occurred after World War II [30, 36], and the name remains in the present classification of education. Subsequently, many departments of home economics were designated as dietitian training courses in Japan after 1950 [30, 37], and dietitian courses introduced PHN as a subject in response to the growing importance of applied nutrition in 1973 [16, 18, 37]. In the present study, all the PHN programs we identified were training courses for dietitians or registered dietitians (data not shown). However, the development of PHN research in the field of home economics might be hampered by the fact that home economics is mainly focused on food and cooking, and not on human health [20]. A previous study recommended that the field of nutrition science requires independent recognition, in other words disentanglement from home economics, so that it can offer specializations such as dietetics or food technology, and that PHN should be identified under the larger rubric of public health science [12].

Our finding that the number of PHN-related articles per PHN program was lowest in Japan suggests the presence of problems in the ability of Japanese education to foster researchers in PHN. Although curricula and educational systems of PHN among the states should be further investigated, university curricula for PHN in Japan have been previously criticized. In dietitian training courses, most learning time in PHN is spent on administrative science such as related laws and regulations, and less time is allocated to the diagnosis of current issues or programme planning for nutrition promotion at a public scale [17, 18], making these courses less attractive for dietetic students. Further, a lack of teachers with sufficient skill to teach nutritional policy and management has also been noted [17]. Additionally, dietetic students are required to pass various subjects in a national examination, such as clinical nutrition and food service management [38]. Overall, it appears difficult to study PHN in any depth in the limited time available. South Korea has a number of graduate schools of public health that are modeled after schools of public health in the United States, and offer PHN education [39, 40]. This may account for our finding that South Korea produced the highest number of PHN-related articles per PHN program among all the states. Postgraduate programs are suitable for further PHN learning and are important in fostering skilled and competent professionals for PHN [7, 8, 11, 14]. Thus, reconsideration of the educational contents and systems for PHN at both undergraduate and graduate school levels may be required for the development of human resources for PHN in Japan.

As similarly found in a previous report on human nutrition [19], educational institutions with PHN programs produce fewer articles than those without a PHN program in Japan, South Korea, and Taiwan. Although the fundamental cause of this inconsistency is unknown, one possibility might be differences in research environment or skills between those educational institutions with and without a PHN program. Such differences might also result in differences in the quality of research education.

Our finding that Japan had the lowest number of PHN-related articles per PHN program might be explained not only by PHN education but also by research resources. One obstacle to PHN research in Japan is the low availability of data derived from government statistics. The use of data from the Japan National Health and Nutrition Survey is burdensome, requiring many procedures including consultation, usage application, and payment of a utilization charge to the Ministry of Health, Labor and Welfare, etc. [41]. In contrast, data from national nutrition surveys in South Korea, Taiwan, and China are available from the website for free with no or few application procedures [42–44]. South Korea had the highest proportion of PHN-related articles published by hospitals to total number of articles, and 78% of the articles published by hospitals used data from the Korea National Health and Nutrition Survey (educational institutions: 31%, research institutes: 25%). Thus, wider availability and easier access to Japanese government statistics might aid the promotion of PHN research.

This study revealed a number of issues in education and research of PHN in Japan. Japan now faces the problems of super-aging, as well as various health problems such as an increase in lifestyle-related diseases [45]. Accordingly, the importance of PHN practice is set to grow in Japan. Since an insufficient number of professionals or insufficient competency can be barriers to research and strategies of PHN [46], a stronger emphasis should be placed on the development of specialists in this field. At the same time, it is also necessary to promote PHN research aimed at generating evidence for PHN practice for Japanese. This will require further investigation into the quality of education and research environment of PHN.

The major strength of this study was its cross-country comparison of education and research activities in the field of PHN in East Asia. Our results provide fundamental information to understand the current status of education and research of PHN in Japan, South Korea, Taiwan, and China. Moreover, this survey was designed and conducted by researchers familiar with the language and education system of each country and region, in such a way that a systematic investigation would be performed in a unified manner as possible.

Several limitations of this study should also be acknowledged. First, there might be other classes related to PHN besides those surveyed in this study. Indeed, it was difficult to identify PHN classes due to the large variety of definitions of PHN [47]. We investigated classes whose name included the vernacular word that directly meant PHN. However, this might have led to underestimation of the number of PHN classes, although not to overestimation. Further, only departments which required PHN class as a compulsory subject were regarded as PHN programs, and this might also have resulted in the underestimation of educational institutions of PHN. In South Korea, we identified 87 departments that had PHN as an optional subject. However, we did not include optional PHN courses as it was difficult to determine whether the students in those departments actually took a PHN class. Second, PHN education at graduate schools was not investigated. However, given that PHN programs are mostly offered at the undergraduate level in Japan, we considered that a focus on college and university education was reasonable. Third, we could not unify the survey methods for PHN education in China with those of Japan, South Korea, and Taiwan, due to limited data availability. Project 211 was limited to national schools, which account for only 9% of all colleges or universities in China. Moreover, we could not obtain information from some of the colleges or universities, due to the lack of a contact address or response from the school. Therefore, our results do not represent the current situation in China. Finally, it is likely that PHN-related articles are published in journals other than *Public Health Nutrition*. A variety of journals are likely to publish PHN-related articles in the field of nutrition, medicine or other. However, given the scope of the journal as well as the fact that there are no established criteria for defining a PHN-related article, inclusion of a wide range of relevant journals in addition to *Public Health Nutrition* is unlikely to lead to any markedly different conclusion.

## Conclusions

This study provided preliminary information on the current status of education and research of PHN in Japan compared to that in South Korea, Taiwan, and China. Japan had much larger number of departments with PHN and fewer publications in the field of PHN compared with those in the neighboring countries. For the development of PHN, the quality of education and research environment may deserve further investigation.

## Abbreviation

PHN: Public health nutrition
